# Management of Harlequin Ichthyosis: A Brief Review of the Recent Literature

**DOI:** 10.3390/children9060893

**Published:** 2022-06-15

**Authors:** Maria Tsivilika, Dimitrios Kavvadas, Sofia Karachrysafi, Antonia Sioga, Theodora Papamitsou

**Affiliations:** Histology and Embryology Department, Faculty of Medicine, Aristotle University of Thessaloniki, 54124 Thessaloniki, Greece; tsivilikamaria@gmail.com (M.T.); kavvadas@auth.gr (D.K.); sofia_karachrysafi@outlook.com (S.K.); sioga@auth.gr (A.S.)

**Keywords:** ichthyosis, Harlequin ichthyosis, keratinization, surgical management, treatment

## Abstract

Harlequin ichthyosis (HI) is a life-threatening genetic disorder that largely affects the skin of infants. HI is the most severe form of the autosomal recessive disorder known as ichthyosis. It is caused by mutations in the *A12* cassette (lipid-transporter adenosine triphosphate-binding cassette *A12*). Neonates affected by this disease are born with specific morphological characteristics, the most prominent of which is the appearance of platelet keratotic scales separated by erythematous fissures. The facial features include eclabium, ectropion, a distinct flattened nose, and dysplastic ears. A common finding among those with HI is impaired skin barrier function. The purpose of the present narrative review is to assess the most recent literature regarding the management of HI. Emphasis is given to surgical management and consultation, to the indications for timing and surgical intervention, to the risks that are presented with surgery, and to the details of the surgical procedure itself. Management of HI requires a multidisciplinary team of experts, and specific guidelines are needed in order for the risks to be minimized and viability to be increased.

## 1. Introduction

The pathological condition known as Harlequin ichthyosis (HI) is a severe but extremely rare form of congenital ichthyosis. This disorder affects significantly the process of keratinization. During the keratinization procedure, the epidermal skin layer differentiates to form the stratum corneum [1,2,3,4]. Epidermal keratinization begins approximately between the 20th and 24th week of the gestational period [5]. Thus far, it is known that HI is caused mostly by the truncation of *ABCA12* gene mutations [6]. This specific gene is responsible for the regulation of lipid-transporting proteins in the epidermis. Mutations cause severe imbalances to the homeostasis of the stratum corneum [7]. Scientists are making efforts to identify all the possible mutations that are responsible for the clinical manifestation of HI. A recent case study suggested that the phenotype of HI is specifically determined by the novel *ABCA12* mutation, *p.Gly-1508Val* [8]. Another recent case study found novel deleterious intronic variants that could lead to the most severe manifestations of the disease [9].

The prevalence of the disorder is approximately 1 out of 300,000 infants [4]. There is no significant association between sex and race with the incidence of HI [4]. Until recently, fetuses affected by HI were either stillborn or died as newborns a few days after birth. Thankfully, in the last two decades, the use of advanced therapy protocols, such as retinoids and novel procedures, has allowed several infants to exceed this narrowed period of time and survive longer [1,2,3,4].

There are three distinct subtypes of HI (Table 1). The differentiation among these subtypes is based mostly on the reported skin abnormalities. The abnormalities on the lamellar structure of the epidermis are caused not only by the dysregulation of the lipid transportation but also by the severe desquamation due to the hydrophobic nature of the lipids. These anomalies are caused by the loss of the normal function of the *ABCA12* gene [10,11]. The skin is sensitive to epidermal “fractures” and to excessive fissures’ formation due to the significant lack of connective substance [10,11].

The first common abnormality is the irregular formation of the lamellar granule structure, which appears as a shortness of aberrant granules. Additionally, lipid droplets are detected in the vacuoles and the stratum corneum. The abnormal lamellar form can lead to extreme water reduction due to the hydrophobic properties of lipids. Another common irregularity is the abnormal conversion of profilaggrin to filaggrin. This atypical conversion leads to the excessive production of filaggrin and the inability to form a strong keratin complex. Other severe irregularities entail the expression of keratin and the detection of keratohyalin granules, either regular or irregular [11,12,13,14].

The abovementioned abnormalities can negatively affect the desquamation process and lead to the distinguish characteristics of the disease; an armor-like thick and fissured stratum corneum [14]. Further evidence suggests that calcium may be related to the development of HI because of its significant role in the differentiation of the epidermis [14,15]. Calpains are a distinctive form of proteolytic enzymes, activated by calcium. There is indication that these enzymes are essential for the signal transduction processes during differentiation [14,15].

The literature reports a variety of symptoms that are related to HΙ. The most prominent and conspicuous ones are responsible for the name of the disease: brown or white skin, grooved or cracked, eclabium, ectropion, nasal hypoplasia, lack of external ears, sparse scalp hair, short limbs, hypoplastic digits, and complete absence of eyebrows and eyelashes [13,14]. Hypoventilation, respiratory failure, and respiratory depression are also reported among the symptoms, attributed to the confinement of the pectoral wall by the armor-like skin. In addition, there are central nervous system traits of the disease, such as seizures, that raise concerns [16]. Due to the cracked skin, infants also suffer excessive dehydration which can lead to severe metabolic dysregulation. Finally, infants face significant feeding problems and gastrointestinal disorders, leading to malnutrition [16].

A detailed family history record is quite valuable, as it could lead to the identification of a variety of possible risk factors such as genetic reports, siblings affected by the disease, consanguinity, and history of other abnormalities that affect keratotic skin [17]. The genetic pathology of Harlequin ichthyosis has been well known in the literature for several years and has also been shown in the official classification of inherited ichthyoses [17]. A common clinical method used for the diagnosis of HI is ultrasonography [18]. Diagnosis can also be made by either 2D or 3D ultrasonography according to the manifestation of specific symptoms (i.e., cystic formation in front of the eyes, limb anomalies, and hypoechogenic amniotic fluid). Until the identification of the *ABCA12* gene, a fetal skin biopsy was the preferred method of prenatal diagnosis, performed at the 20–24th week of gestation. It should be noted that light microscopy may also give a falsely negative outcome during the interpretation of the biopsy samples, based on the sites of the tissue collection [19,20]. Nowadays, the standard technique for the detection of HI is next-generation sequencing (NGS). Nevertheless, clinical investigation and ultrasonography are, in most cases, adequate and, therefore, necessary for a safe diagnosis [21].

Regarding the surgical management of HI, the case studies presented in the literature are limited. Surgical cases offer valuable knowledge, focusing on digital ichthyosis, a common manifestation among HI patients, which threatens the neurovascular integrity of the upper limbs [6,22]. Circumferential release and subsequent Z-plasty reconstruction result in beneficial outcomes in cases of congenital constriction bands [6,22]. It is essential to assess the surgical management of HI and, therefore, in this review, emphasis was given to surgical reports.

Apart from the management of the disease, parents should be given appropriate support by social workers and psychologists. As most infants do not survive the neonatal period, it is crucial for the family to cope with the possibility of their probable loss [23]. In addition to the NICU (Neonatal Intensive Care Unit), the infant has to face a number of surgical procedures, such as reconstructive surgery and skin grafts, in order to manage the skin injuries and to treat the extremities and the eyelids [2,24]. Inevitably, the infant’s quality of life will be seriously affected due to the constant medical follow ups and medication intake [25,26]. There is also high probability for these children to develop psychological disorders, mainly due to the fact of their appearance [25,26].

The aim of this study was to analyze the options offered by the current literature regarding the management of HI cases. Emphasis is given to the surgical management of newborns. Because of their unique characteristics, these patients demand specialized care and multidisciplinary physicians’ attention.

## 2. Materials and Methods

This review aimed to assess the current literature regarding the medical and surgical management of HI. As the disease has an extremely rare frequency and high mortality rates, the management process can be a challenging procedure for the physicians, the rest of the medical team, and the infant’s family. Meticulous research was performed using the publicly available internet databases PubMed and Scopus. The key words used for the investigations were “*harlequin ichthyosis management*”, since 1980; there where 97 articles available.

**Figure 1:**
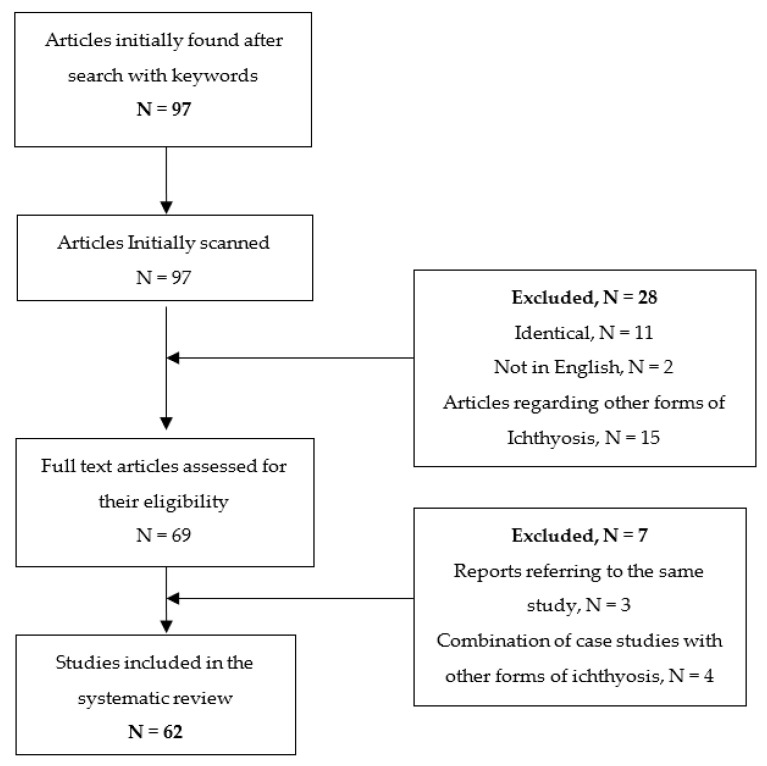
PRISMA flowchart of the selection of the studies.

The research’s results are depicted in a PRISMA flowchart (Figure 1).

## 3. Results

Admission to a specialized health center with a NICU (Neonatal Intensive Care Unit) for newborns with HI is highly recommended [27,28]. Intensive care for the infant includes a multidisciplinary team of experts that consists of nursing personnel, physical therapy specialists, an orthopedic team, plastic surgeons, ophthalmologists, otolaryngologists, geneticists, dermatologists, and neonatologists. Due to the high morbidity ratio associated with the disease and the elevated risk of respiratory failure, direct intubation was required in most cases. Apart from the complications caused by the infant’s immature growth, actions should be taken to prevent electrolyte imbalance, respiratory distress, malnutrition, and infection. All the above precautions increased the survivability ratio and were optimally performed in the NICU [27,28].

In a recent cohort study, the age range of HI patients who survived the early neonatal period ranged from 10 months to 25 years. The average survival rate of the disorder was approximately 55% [6]. HI’s highest fatal rates were reported in the first three months after birth due to the fact of respiratory failure or sepsis (75% of the cases). One-third of the patients developed recurrent skin infections, and a significant percentage (44%) could not maintain normal weight [6].

The literature suggests that affected neonates should be maintained in an incubator with additional humidity, according to each patient’s needs [29,30]. Constant monitoring of the kidneys and liver is necessary. Urine output, daily weight, and serum electrolytes should also be monitored. One of the most significant factors for increased survivability during the neonatal management was the prevention of infections. In addition, thick scales and deep fissures can perforate the epidermal skin layer and lead to significant discomfort and ache. Therefore, the majority of neonates require narcotic pharmaceuticals. It should be noted that not all patients manifested strong phenotypes, and their skin soreness or ache was manageable [29,30].

A tricky pathophysiological factor is the skin barrier dysfunction due to the surface-to-weight ratio, which is disproportional in comparison to the normal [31]. Skincare should be included in the treatment modalities. Infants should undergo such treatments one or two times per day in order to promote the shedding of the stratum corneum and increase hydration [32]. A common complication of HI is also the necrosis of the digits. In these cases, surgical interventions are recommended. The literature describes several fasciotomy techniques including an incision technique of the linear band. The application of topical retinoids, such as tazarotene cream at a 0.1% concentration, and the soft splinting of feet and hands can also act as an alternative therapeutical intervention in order to avoid surgery [19].

Increased skin turnover raises the caloric demands of the infant. However, jaw constriction and eclabium could interfere with the normal oral feeding [2,33]. Neonates of this category often require nasogastric or oropharyngeal tube feeding. Once sucking and swallowing functions have been adequately performed by the infant, breastfeeding could be used to further strengthen the bondage between child and mother. The literature also reports cases where inadequate suck capabilities led to the inability to maintain caloric needs and, consecutively, to supplemental tube feeding. Cases of frequent emissions were also reported and were associated with gastrointestinal dysmotilities [2,33].

Deficiency in vitamin D and cases of rickets have been observed in HI; however, there is conflicting evidence regarding whether a vitamin D deficit is caused due to the fact of pharmacological interventions such as oral retinoids [34]. Moreover, lower levels of vitamin D in neonates have been correlated with the risk of sepsis. The most recent studies suggest that skin scaling is responsible for the significant decrease in vitamin D levels [35]. In addition, several patients had elevated parathyroid hormone levels [35]. The above facts suggest that continuous monitoring of vitamin D and possible supplementation may also be necessary for the management of an HI infant [34].

Around the eyelids, the stratum corneum appears considerably thicker, a situation that leads to the bilateral ectropion. Infants are at a higher risk for developing strabismus, conjunctivitis, and keratitis. Even though the recommendations for eyelid treatment include the application of ophthalmic ointment upon the eyelids margin, surgical interventions have also been reported [36,37]. More specifically, surgical interventions focused on the full-thickness autografts from engineered human skin and from the posterior auricular skin. There is no evidence that early surgery decreased the possibility for ectropion in a timeframe of 6 to 12 months [36,37].

Furthermore, the circumferential plaques (polycyclic erythematous plaques) must be considered as a sign to perform early plastic surgery consultation [38]. Even though polycyclic plaques that are nonconstrictive do not pose a significant threat of tissue perfusion, intravenous fluids should be administrated in order to increase pressure within the noncompliant section, constrained by the circumferential plaque. An early consultation by a plastic surgeon is necessary in order for a close relationship with the infant’s family to be established and facilitate timely interventions [38].

However, the surgical procedure is not without risks. More specifically, surgical wounding increases the possibility of infection, one of the primary risk factors for neonatal morbidity [31]. The literature recommends the use of antibiotics, topically applied, until the surgical wounds are fully healed [37,38,39]. Indeed, a recent case reported that implementation of topical antibiotics prevented completely the infection of the surgical wound [39]. Apart from infection, the surgical procedure also poses as a significant risk factor for iatrogenic injury. This possibility of harm is greater in the upper limbs due to the fact of their structure, slippery texture, and immature size, which consecutively makes immobilization challenging [40]. Sedation before surgical procedure is necessary for decreasing patients’ movement during the intervention. However, the odds of oversedation and the augmented need for intubation increase the procedure’s risk factors [40]. Infants with HI have delicate and slippery skin, which increases the difficulty of the intubation process and limits the tube’s secured implementation. Initial procedural sedation is usually necessary to reduce the patient’s discomfort and movement [41,42]. Nevertheless, the surgical team should assess the benefits of procedural sedation at the time. If no apparent benefits are reported, the surgical team may switch to a local anesthetic during the second operation. Recent reports state that local anesthesia was adequate, and the additional risk of the procedural sedation should not be justified [41,42].

Studies have also reported that the use of retinoids (e.g., trifarotene) demonstrate significant tolerability and could treat acne and congenital ichthyosis [43]. Other studies suggest through specific cases that the use of acitretin inhibits wound healing [10,44]. In these cases, there were significant difficulties during the regeneration and recovery of large surgical wounds [10,44]. Another case suggests that systemic retinoids administration might have beneficial auxiliary therapeutic effects on the reparation of eyelid anatomy in HI cases [45]. Therefore, despite the possibilities of retinoids’ protective effects on ischemic injury, there are studies that do not suggest retinoid therapy in infants and recommend not to preclude them to a surgical intervention [38]. A recent novel randomized controlled trial study proposed vitamin D intake as a promising alternative for managing congenital ichthyosis in comparison to acitretin [46]. In 2021, the scientific community published a consensus of recommendations regarding the safety of the topical and systemic administration of retinoids [47]. Disorders such as HI demand the intake of these drugs from early childhood, and this endangers patients not only due to the already known adverse outcomes on the eyes and bones but also on the less studied side effects such as psychiatric disorders or cardiovascular diseases [47]. Hence, guided practices on retinoid administration or even alternatives, such as vitamin D, should be considered.

Regarding surgical techniques, the positioning of the infant is also crucial. HI patients present significant difficulties in terms of vascular access and securing an airway. In most cases, the lateral decubitus position is selected, and asymmetric incisions are made on the extremities. If adequate anesthesia is required, it could be provided by lidocaine with epinephrine at a 0.25% concentration. The maximum dosage follows conventional guidelines (7 mg/kg). In more complex cases, where all four limbs require release, the surgical team should assess whether to stage the procedure or not [36,48,49].

The most beneficial technique is constriction release without violating the total thickness of the underlying dermis [50]. The depth of the incisions should be adequate for the visible release of the constriction. Surgeons can provide hemostasis with many techniques, the most prominent of which is electrocauterization due to the fact of its specific advantages, using a monopolar grounding pad that does not require skin attachment [50].

There is a lack of adequate knowledge regarding the management of encased or constricted limbs in patients with HI. This lack of knowledge increases the difficulty of decisions making regarding surgical intervention in a constricted limb [51,52]. There are reports of tissue necrosis and digital autoamputation; however, the percentages of the outcomes in unoperated individuals remain unknown [51,52]. Therefore, parents should be informed that without surgical release, there is the possibility of bilateral digit necrosis, without excluding the probability of spontaneous recovery. The surgical team and the patient’s parents should agree on the associated risks and the probable odds to salvage the extremities, especially in patients with skin abnormalities that make them unlikely candidates for prosthetic rehabilitation [52,53,54]. Increasingly, studies report that surgical interventions in the early stages managed to save the limbs in patients with ichthyosis. The above cases emphasize the utilization of multiple Z-plasty transposition flaps that effectively reorient and lengthen the scar lines to perform tension-free closure. This method decreases the risks of the future formation of constrictive bands [6,22,51]. Other case studies focus on the surgical management of cicatricial ectropion, a common ophthalmic feature of HI, leading to corneal exposure and lagophthalmos. Even though the surgical correction of cicatricial ectropion has been reported as quite challenging and the results unsatisfactory [55], proper processing of the donor and the recipient site before surgery makes the grafting procedure more accessible and successful, with increased survival rates [56]. The need for reoperation for dealing with recurrent ectropion has been reported from several studies [55]. Another surgical case of HI reports that the uptake of neotigason prior to and after the surgery (20 days prior), followed by topical administration of acitretin (or tazarotene), did not manage to prevent or delay the reoccurrence of ectropion. Further studies are needed on this matter [57].

Hands may not require treatment by the time of the lower extremity release, but this depends on the stability of their appearance. Moreover, the operation of both hands and feet is problematic because of the inadequate knowledge regarding the amount of local anesthetic that can be safely administrated [19]. Especially for neonates that are usually underweight, anesthetic administration can be a serious predicament [19]. In addition, the total encasement of the hands because of plaque formation can make the case even more challenging in comparison to the feet, which are typically swelled distal [22,58]. Tissue ischemia on fingertips is usually evident upon physical examination, and by that time, the injury is irreversible. Since early ischemic cases are difficult to diagnose before they become irreversible, a low threshold is recommended to assess the release of encased or constricted limbs. The earliest signs of discoloration and progressive firmness of the tissues should be considered as indications of eminent compartment syndrome [22,58]. A recent case report made an attempt to provide valuable knowledge for managing patients with HI regarding the anesthetic methods [59]. The authors of the abovementioned case suggest the use of a novel protection dress for the skin of the child, alongside the use of an antimicrobial dressing [59]. This combination forms an ideal protection barrier for the patient during the procedure [59]. Finally, another report managed significant improvement on the hands of an HI patient [60]. In this case study, the management was mainly achieved through a full-thickness graft of skin. Before the surgical procedure, the patient was on retinoid therapy (i.e., acitretin) and topical treatments [60].

## 4. Discussion

HI is a severe but rare disorder that is fatal for newborn infants. In order to deal with this unique medical challenge and increase the survival rates, a multidisciplinary team of physicians, among other experts, is essential. Researchers have already focused on the genetical aspect of the disease. Despite knowledge of the responsible gene, the mutations present several disparities among each case, leading to a variety of symptoms and affecting the survival rates. Utter comprehension of *ABCA12* gene mutations is vital for managing the disorder. The pathophysiological mechanisms responsible for the disease’s manifestation are associated with the mutations created in the genome and, therefore, researchers suggest that this knowledge will increase the survival rates after birth [6,7,8,9].

The affected patients will face several challenges, especially during the neonatal period. In order to deal with this dire situation, genetic investigation is not enough. It is essential for physicians to have at hand effective guidelines, confirmed by the international literature. As time is of the essence, proper instructions could make the difference. The limited literature resources for HI led physicians to the implementation of managing methodologies and treatments from other ichthyosis cases or even similar dermatological disorders. In regard to surgical preparation and management, there have already been many approaches as described in Section 3 of the present review. Admission to an NICU should not be avoided and increased humidity is essential. Applying topical retinoids has been suggested as alternative to surgery but with no satisfactory results in most cases. Moreover, their combination with surgery did not manage to control the relapse of the disease. Alternatives, such as vitamin D intake, should be considered in order to avoid the retinoids’ side effects and investigate further the possibility of better outcomes.

Limb encasement and constriction may also lead to autoamputation due to the presence of tissue necrosis. Therefore, confirmation of any possible limb confinement should be a reminder for surgical assessment. Operation using local anesthesia mitigates risk related to airway obstruction; however, parental counseling before the surgical procedure should include information regarding possible complications, due to the possibility of infections, and other recovery plans.

Nowadays, novel research focuses on the immune system and the full characterization of the immunological profiling of these patients, as it may lead to therapeutic approaches that have not yet been implemented [61]. Other skin disorders, such as psoriasis and atopic dermatitis, share the same immune characteristics as HI. For instance, the immunoactivity is similar to the allergic reactions including IL-17 signaling. The scientific community should investigate further the possibility of immunotherapy, such as monoclonal antibodies.

Another novel perspective is the topical application of enzymes that are involved in desquamation [11]. Kallikrein proteases were found on experiments with mice to mitigate the hyperkeratosis when topically applied. This evidence suggests that the lack of proteolytic enzymes due to presence of *ABCA12* gene mutations could be the main culprits in the pathophysiology of HI.

Cooperation with parents is also essential in order to avoid serious predicaments [62]. It is vital for the survival of the infant that parents understand the situation and the possible options (both pharmaceutical and surgical) in order to avoid behaviors that may endanger the child further.

At the moment, the most common surgical interventions are autologous skin grafts. However, because of the high possibility of sepsis and bacterial infections in the epidermis and the skin layer, invasive procedures should be treated with extreme caution. On the other hand, for some cases, such as nasal occlusion, endotracheal intubation is unavoidable [22,58]. Therefore, it is an undeniable fact that HI surgical and general management can be quite challenging and specific guidelines will make the difference.

## Figures and Tables

**Table 1:** Main types of Harlequin ichthyosis.

Type of HI	Keratin Expression	Keratohyalin Granules	Profilaggrin	Filaggrin
I	Normal	Normal in appearance	Yes	No
II	Types K6/K16 present, types K1/K10 reduced	Small and round like, anomalous	Yes	No
III	Normal	Absent in interfollicular epidermis	Expressed in intradermal sweat ducts, barely present in interfollicular epidermis	No
